# Stephen C. Stearns and Ruslan Medzhitov, Evolutionary Medicine

**DOI:** 10.1093/emph/eow008

**Published:** 2016-02-28

**Authors:** Gilbert Omenn

**Affiliations:** University of Michigan, USA

This book is a gem of a textbook about the principles of evolutionary thinking and the relevance of evolutionary biology to clinical medicine. The authors draw upon decades of experience teaching medical students at Yale, with interesting examples, including experimental observations in other species. Their format with a detailed table of contents and cogent summaries within each chapter is helpful.

Every trait/every organism has a history. A transposon insertion enabled the emergence of the vertebrate adaptive immune system with antibody diversity. Invasive placentas carry the tradeoff risk of metastasis by cancers of glandular tissues. Some genetic variations are associated with specific infectious disease risks. In pharmacogenomics, copy number variants of CYP drug metabolizing genes guide drug developers to design drugs around such highly variable human traits. Mismatches of species inheritance and modern environments test ‘reaction norms’ and lead to risks of metabolic syndrome and related disorders, though the relevance of sterotyped ‘paleodiets’ is challenged in the closing chapter. Allocation of energy probably explains tradeoffs of number of offspring versus survival of individuals.

Early chapters draw the interest of students/readers with the themes ‘What is a patient?’ and ‘What is a disease?’ Humans have an unusual life history in several traits with medical significance. Our infants are large, fat and relatively underdeveloped at birth, compared with chimps, with brain size increasing for 7 years and large adolescent growth spurts, three years earlier in girls than in boys. Chimps do not have menopause. The authors seem dubious about the ‘thrifty genotype’ and ‘thrifty phenotype’ hypotheses. They note associations with reported changes in microbiomes, including autoimmune disorders. Patients are mosaics of traits of different evolutionary ages and bundles of tradeoffs with plastic responses to their current environments. In classifying diseases, they properly emphasize genotype × environment and gene × gene × environment interactions. They recognize the complexities of diseases that are by-products of defense systems or homeostasis or maternal-paternal conflicts. An overarching principle is that most diseases have both mechanistic and evolutionary explanations. Such an analysis enriches our thinking about disease and disease prevention, with inflammatory bowel disease and the vulnerability of neurons, myocardium, and kidney as terrific examples.

The characteristics and costs of our evolved defenses constitute a particularly intriguing chapter, embracing the interrelations of homeostasis, maintenance and constitutive and inducible defense mechanisms of many types. The examples are current, including proteosomal, lysosomal and autophagocytic removal of damaged proteins and lipids; degradation in these mechanisms is associated with various processes of aging. Of course, the evolutionary logic of a particular cost-benefit ratio for a defense mechanism may now be obscure because it was determined long ago for environmental exposures and physiological priorities of that era, as illustrated with starvation, dehydration, extreme heat and cold, predators and pathogens.

Subsequent chapters address pathogen and symbiont exposure, cancers (we should always use the plural), reproductive medicine and mismatches, and mental disorders and drug addiction (important but poorly characterized phenotypes). The closing chapters are particularly useful in stimulating deeper consideration of the need to understand cross-consequences of interventions for individual health and population health, and by raising ‘open questions’ that should help readers and observant clinicians to expand the applications of evolutionary medicine, thereby enhancing medicine and public health. For example, in the important context of bacterial resistance, they highlight the potential usefulness of bacteriophage therapy, of siderophore quenching and quorum sensing (recently published mechanisms which have received international awards in the past 2 years from EMPH and from Shaw Prize of Hong Kong), and of late-acting insecticides (which might be applicable to Aedes mosquitoes in the current outbreak of Dikavirus in Brazil and to malaria more broadly).

Referring to the flipped classroom and active learning, Stearns and Medzhitov have made You Tube and iTunesU presentations for student on-line access under ‘Stearns Evolutionary Medicine’, currently 65 videos, aimed at stimulating classroom discussion and exploration of the cited literature and other sources. General readers will find this book quite stimulating, too.

